# All-Natural Moss-Based Microstructural Composites in Deformable Form for Use as Graffiti and Artificial-Porous-Material Replacement

**DOI:** 10.3390/ma15249053

**Published:** 2022-12-18

**Authors:** Bu-Gon Kim, Min-Ho Yoon, Jaehwan Kim, Jung-Hwan Oh

**Affiliations:** 1 Department of Mechanical Engineering, Wonkwang University (WKU), 460 Iksan-daero, Iksan-si 54538, Jeollabuk-do, Republic of Korea; 2 Department of Mechanical System Engineering, Kumoh National Institute of Technology (KIT), 61 Daehak-ro, Gumi-si 39177, Gyengsangbuk-do, Republic of Korea

**Keywords:** sound-absorbing materials, green/eco composites, composite structures, mechanical properties

## Abstract

Although artificial porous materials are useful for dissipating acoustic waves, they pose a major environmental threat as most are non-recyclable. Developing sustainable structural materials with the mechanical and energy-absorption properties required to replace artificial porous materials is currently a key challenge. Here, we report, for the first time, a novel microstructure using all-natural moss with a compressive strength of up to 2.35 GPa and a sound-absorption performance of up to 90%, depending on the additives, such as yogurt, starch, and beer. In addition, the moss-based microstructure was applied as graffiti to a three-dimensionally printed house model to demonstrate improved performance against the effects of sound. By incorporating energy-absorbing materials without harmful substances, the desired structure can be decorated with the graffiti method. This work could pave the way for attenuating sound-wave and impact noise by using graffiti work on structural composite materials.

## 1. Introduction

Noise pollution, a public health hazard, is regarded as a major obstacle to quality of human life. In particular, noise and vibration can directly endanger human health, causing insomnia, vibratory tinnitus, neurotic and heart diseases, and even mental disorders [1,2,3,4,5]. Therefore, various sound-absorbing structures based on resonant and porous sound-absorbing materials are considered to control the noise level [6,7,8,9,10,11,12]. However, although resonant sound-absorbing materials have excellent sound-absorption performance in the mid-low frequency range, their drawbacks include the use of rigid and non-eco-friendly materials and a complex processing procedure, limiting their practical applications [6]. Unlike resonant sound-absorbing materials, it is well known that porous sound-absorbing materials, containing cavities and apertures, have high sound-absorption coefficients in the high-frequency range [9,13,14].

Recently, various porous sound-absorbing structures have been introduced, including natural materials, such as cellulose [15,16], cork [17,18], and leaves [19,20], and artificial alternatives such as polyurethane [21,22,23], polyvinyl alcohol [24], polyester [25], and ceramics [26,27]. Porous structures composed of polymers or polymer-nanofiller composites have been widely used for sound-absorbing applications due to their low price and ease of design and fabrication [28]. However, porous materials based on synthetic polymers are not environmentally friendly in that they are difficult to recycle and are not biodegradable. In addition, ceramic-based sound-absorbing materials have been actively investigated in various ways, but they are not used widely owing to their high sintering temperatures, heavy weights, and lack of cost-effectiveness [27]. With recent technological advances, the processing cost of natural fibers has been lowered and their quality has improved. Therefore, biodegradable natural fibers, such as wood [18,29], flax [30], and Kenaf [18,31], are frequently applied as sound-absorbing materials. However, the demand for carbon-neutral and sustainable products means many wood-fiber sound-absorbing materials made from timber are considered environmentally unsustainable. Therefore, it is necessary to use a novel sound-absorbing material with carbon-neutral, sustainable, and biodegradable properties.

Here, we propose to utilize moss as a sustainable and recyclable alternative for sound-absorbing materials. Mosses, which are non-vascular bryophytes without clearly distinguishable leaves and stems, grow on trees and rocks, or in wetlands. Because mosses have no vascular tissue, they grow by creeping across the ground. Their roots do not absorb water, although a tissue called a flat root provides physical support. Instead, a moss can absorb water throughout its entire body, which, if it grows into a lump, resists desiccation. In addition, because mosses do not have a leaf-like cuticle layer to filter foreign substances, they can purify finer grades of dust compared with trees growing in the same area. Mosses exhibit great survivability and expandability with minimal access to water. Here, we focus on the viability and expandability of mosses, as well as their ability to survive dry conditions as they grow into agglomerates.

We applied natural moss as a sound-absorbing material that can be manufactured in any shape. In the form of moss dough, it can be applied as graffiti to the walls of a building, and it can be molded into any desired shape. Moss foams based on raw beer or buttermilk materials have different sound-absorption-frequency ranges, depending on the starch content and the type of raw material. Depending on the starch content, BMA50 (beer-based moss absorber) can tolerate a maximum stress of 2.35 GPa at a strain of 30%. The developed material can be applied in harsh environments and is expected to be compatible with landscaping, depending on the graffiti.

## 2. Materials and Methods

### 2.1. Material Design and Fabrication Strategy

To make a moss-based sound-absorbing material, we prepared natural rat-tail moss, beer (wheat beer, Gompyo, 7br$\ddot{a}$u, Seoul, South Korea), buttermilk (ratio of milk and vinegar 15:1), sugar, and starch. We collected moss, which is common in Korea, and washed and dried it until no water was dripped from it. For the production of beer-based, sound-absorbing moss, 100 g of moss, 60 mL of beer, and 10 g of sugar were mixed in a beaker. First, 0, 25, and 50 g of starch were mixed and then finely ground 3 to 5 times in a blender for 1 to 2 min each. The finished beer-based moss dough was placed in a mold and allowed to dry naturally for 3 to 5 days. To prepare a buttermilk-based sound-absorbing moss material, we first mixed 100 g of moss and 50 g of buttermilk in a beaker. The same manufacturing method as the beer-based sound-absorbing moss material was then followed [32,33]. The morphology of all specimens was observed using a microscope (Eclipse LV-S6PL, Nikon, Tokyo, Japan).

### 2.2. Mechanical Performance

To measure the mechanical stiffness of the developed samples, a compression-cycle test was performed using a universal testing machine (AGX-V, Shimazu, Berlin, Germany). Samples of BMA0, BMA25, BMA50, BMMA0, BMMA25, and BMMA50 were cut to a diameter of 30 mm and a thickness of 10 mm. The samples were placed on a universal-testing-machine compression jig, which was cycled at a speed of 2 mm/min. The energy-dissipation density (∆*E*) of our samples was calculated from the area of stress–strain hysteresis loop using a dynamic cycle process, as given Equation (1) [34].

(1) $$\Delta E=\int_{loading}^{\sigma }d\varepsilon -\int_{unloading}^{\sigma }d\varepsilon$$

### 2.3. Sound-Absorption Performance

To measure the sound-absorption performances of the developed samples, the SAC at frequencies between 125 and 6300 Hz was analyzed using an impedance tube (SW260, BSWA). All samples were cut to diameters of 30 and 60 mm, and measured to have a single thickness of approximately 10 mm. For all samples, the sound-absorption coefficient (SAC) of each sample was measured three times, and the average value of these values was derived. The SAC of each sound-absorbing material was measured three times with an impedance tube and the average value was used for analysis. The noise reduction coefficient (NRC) can be obtained by averaging the sound absorption coefficient (α) at 1/3 octave band frequencies of 250, 500, 1000, and 2000 Hz, as given in Equation (2).

(2) $$NRC=\frac{\alpha_{250}+\alpha_{500}+\alpha_{1000}+\alpha_{2000}}{4}$$

The acoustic activity (α) can be obtained by normalizing the integrated area of sound absorption coefficients by the frequency range, as in Equation (3) [9].

(3) $$\alpha =\frac{\int_{F_{1}}^{F_{2}}\alpha \left(f\right)df}{F_{2}-F_{1}}$$

### 2.4. Impact of Sound-Absorbing Performance

Using three-dimensional printing, we printed a scale model of the actual building on 6 sides. Next, 5.62-g and 45.07-g metal beads were allowed to fall freely from heights of 340 mm and 550 mm, respectively. At 100 mm from the three-dimensionally printed scale model, a sound-lever meter (SC202, CESVA) was used to analyze 1/3 octave band frequencies and measure the resulting noise. The moss house was made by applying a moss paste to the outside of a three-dimensionally printed scale model. The noise test used the same procedures as those applied to the BH model. The gravitational potential energy $\left(PE_{gravitational}\right)$ is for an object near the Earth’s surface, where the gravitational acceleration can be assumed to be constant at about 9.8 m/s^2^. It is calculated as weight times height, as given Equation (4).

(4) $$PE_{gravitational}=mgh$$

After the iron bead falls, the velocity just before the impact can be obtained from the gravitational potential energy and the kinetic energy by energy conservation, as given Equation (5).

(5) $$v=\sqrt{2gh}$$

## 3. Results

The sound-absorption behavior of a porous structural material based on moss is an important factor in the determination of how much noise can be attenuated in a painted wall with moss graffiti in an outdoor environment. To the best of our knowledge, this paper provides the first description of the application of a sound-absorbing material using an all-natural porous material in the form of a foam [20,31,35,36,37].

**Figure 1:**
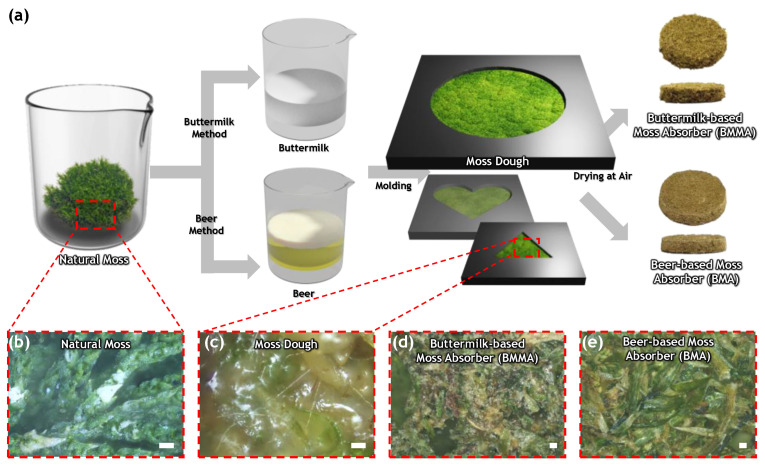
This is a figure. Schemes follow the same formatting. Fabrication and characterization of moss-based microstructural materials. (**a**) Schematic of the manufacturing process for various shapes of porous foam with moss-based dough using environmentally friendly materials of natural moss, buttermilk, and beer. Micrograph of (**b**) natural moss, (**c**) moss dough, (**d**) buttermilk-based moss absorber, and (**e**) a beer-based moss absorber (scale bar: 100 kJ·m^−3^).

To design a free-form structure that adheres to the walls of a structure and that can be molded into round, square, and other brick shapes, we developed a process for kneading moss dough by grinding so that the rhizoids of the moss stick together. Figure 1a is a schematic of a self-assembly method of fabricating beer- or buttermilk-based structural materials from moss. After it was cleaned with purified water, natural moss was dried at room temperature. The moss was then ground in a blender and allowed to decompose so as not to be cut too finely, as shown in Figure 1b,c. With beer or buttermilk as the main ingredient, starch was added to give the moss a porous but solid form [38,39,40]. The final moss paste was then inserted into a frame (including heart and star shapes) to make the desired sound-absorbing material. Because moss dough can be freely transformed, it can be applied to the wall of a building as graffiti, as shown in Figure 1d,e. The self-assembly method can produce a strong, inexpensive, environmentally friendly, and sound-absorbing material in which the non-vascular leaves, stems, and rhizoids of the moss are mixed and stacked on top of each other. This simple and powerful manufacturing method allows the application of graffiti onto any brick, cement, or wood wall, in any desired shape. It is therefore possible to apply aesthetically pleasing sound-absorbing materials and to use them semi-permanently in a sustainable manner.

**Figure 2:**
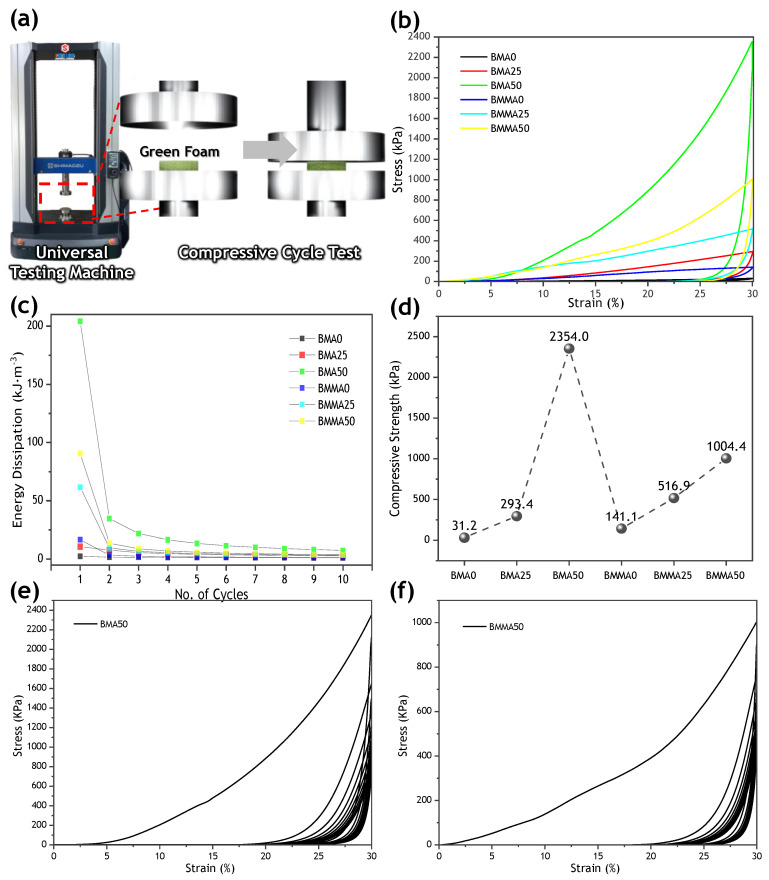
Energy–dissipation characteristics of acoustic energy absorbers. (**a**) Schematic of a dynamic compression cycle test. (**b**) Stress–strain curves of our samples. (**c**) Energy dissipation of the samples over 10 cycles. (**d**) Maximum compressive strength for each sample. Stress–strain curves of (**e**) BMA50 and (**f**) BMMA50 for 10 cycles.

To demonstrate the structural feasibility of a randomly laminated moss-based structure as a sound-absorbing material, the mechanical properties of nature-inspired structural materials were systemically compared with those of composite materials without starch-induced crosslinking [6,7,14,15]. As shown in Figure 2, the compressive strength of the non-starch-treated composite only reached approximately 141.1 kPa, because the moss was clumped together in a finely crushed state. After treatment with crosslinked starch, gelatinization can facilitate the interconnection of the moss; the textural properties of moss-based microstructural composites include strong physical interactions between mosses. Furthermore, the crosslinking area increases with the starch content, forming more randomly arranged and well stacked random structures [16,39]. The beer-based moss material exhibited a relatively high compressive strength of approximately 2.35 MPa, which was more than 75 times greater than that of the starch-untreated composite (Figure 2d). In addition, the BMMA50 (buttermilk-based sound absorber) showed a high compressive strength of approximately 1 MPa. However, the energy-dissipation density of the BMMA50 decreased from approximately 90 kJ·m^−3^ to 4 kJ·m^−3^ over 10 cycles, as measured by a cycle-compression test (Figure 2f). This is because the initially formed moss structure was crushed and collapsed while being compressed to maintain its deformed state.

**Figure 3:**
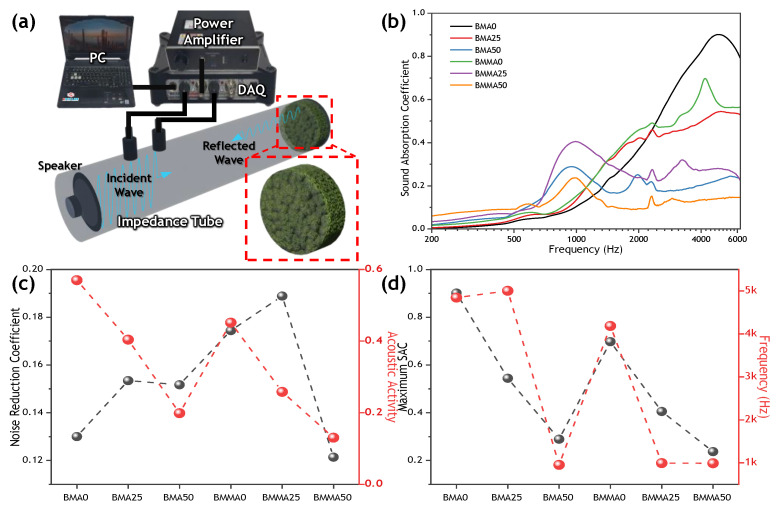
Results of sound-absorption performance. (**a**) Schematic of sound-absorption coefficient (SAC) measurement using an impedance tube. (**b**) Sound-absorption coefficient of the samples. (**c**) Noise-reduction coefficient and acoustic activity of our samples. (**d**) Maximum SAC values and resonant frequencies for each sample.

**Figure 4:**
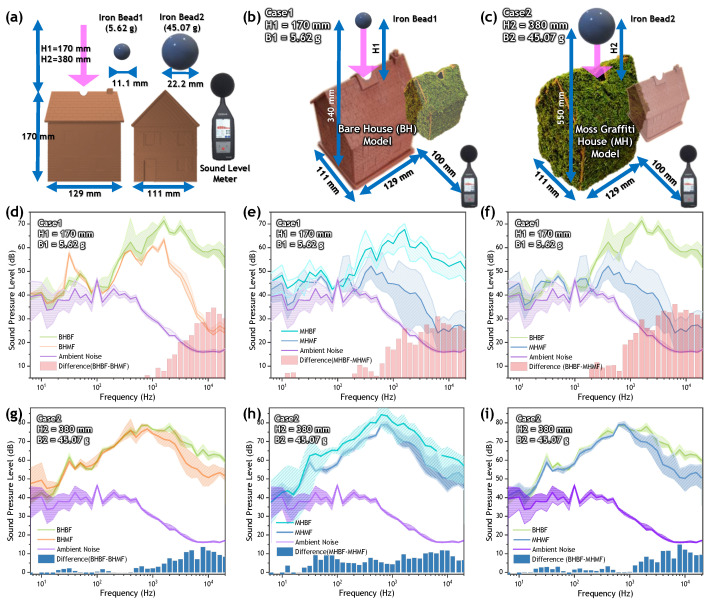
Noise-damping-performance-test results. (**a**) Schematic of three-dimensionally printed house model, two iron beads, and sound-level-meter equipment for noise tests. Experimental design for (**b**) Case 1 and (**c**) Case 2, according to different iron beads and drop heights. (**d**–**f**) Sound-pressure-level—frequency curve of Case 1 for 4 types: BHBF, BHMF, MHBF, and MHMF. (**g**–**i**) Sound-pressure-level—frequency curve of Case 2 for 4 types: BHBF, BHMF, MHBF, and MHMF.

To study the sound-absorption performance of the samples with a thickness of approximately 10 mm, the sound-absorption coefficient (SAC) was measured using an impedance tube (Figure 3a). As shown in Figure 3b, the starch-free, moss-based, sound-absorbing material proved effective in the high-frequency region. However, as starch was added to the sample, the strength of the specimen increased [38,39], and the sound-absorption performance of the specimen shifted its peak frequency to the low-frequency region. The sample of BMA0, a beer-based, sound-absorbing moss material, achieved 90% sound-absorption performance at close to 4.8 kHz, but as starch was added, the sound-absorption performance was lower over the entire frequency band as it changed to a rigid structure, as shown in Figure 3b. The buttermilk-based, sound-absorbing moss material tended to have a slightly weaker sound-absorption performance than the beer-based sound-absorbing material, but as starch was added, a sound-absorption peak close to 1 kHz appeared. The sound-absorbing properties mentioned above indicate that our natural moss composite can replace existing polyurethane sound-absorbing materials as a new structural material [37,40]. The noise-reduction coefficient (NRC) of each specimen was obtained by averaging the values of the SACs corresponding to the 1/3 octave band frequencies of 250, 500, 1000, and 2000 Hz (Figure 3c). In the case of the moss-based sound-absorbing material, all the specimens were manufactured to a thickness of approximately 10 mm. At this thickness, the BMMA25 achieved a NRC value of 0.189. The acoustic activity of a moss-based sound-absorbing material can be determined by the region below the SAC normalized to the entire frequency range. The recorded SACs for the pure BMA0 and BMMA0 without added starch were 0.57 and 0.45, respectively. As shown in Figure 3d, when the maximum SAC of the specimens was compared with the corresponding frequency, the resonant frequency appeared at a frequency of approximately 1 kHz as the compressive strength of the specimen increased. By comparison, the BMA0 and BMMA0, which had low compressive strengths, had higher SAC values, of 0.9 and 0.7, respectively, at high frequencies, of approximately 4.8 kHz and 4.1 kHz. In general, noise is classified into air noise, propagating into airborne noise, and structure-born noise, generated by impacting structures. In this study, Figure 3 confirms the sound-absorbing performance of the sound-absorbing material and can be regarded as a kind of airborne noise. Since the sound energy generated from the structure was transmitted to the air and the structure at the same time, as shown in Figure 4, a scale-model experiment in which moss sound-absorbing material was applied in the form of graffiti on a building model was proposed.

As shown in Figure 4, the moss-based sound-absorbing material we developed can be applied to any type of wall surface as graffiti. Inspired by the study of the effect of façades on an urban acoustic environment, the acoustic environment was measured by measuring scale models [41]. Regarding the test method, the lightweight-impact sound test conducted according to KS F2910-2 was modified to fit the porous-material test. After applying an impact to the floor using an impact ball or a bang machine, the impact sound is measured with a sound level meter. In our study, a miniature model of a house was made using three-dimensional printing, and the impact of the sound produced when an iron bead was dropped from a certain height was measured with a sound-level meter. As shown in Figure 4a, the noise-damping ability was measured using a house model with a width, length, and height of 111, 129, and 170 mm, respectively, a sound-level meter, and two iron beads. Using a bare-house (BH) model without moss and a moss-graffiti house (MH) model with moss on six sides, as shown in Figure 4b,c, iron beads with masses of 5.62 g and 45.07 g were freely dropped from a height of 170 mm or 380 mm from the roof of each house model. The gravitational potential energy (PE) of a 5.62-g iron bead free-falling from 0.34 m was 18.75 mJ, and the velocity at impact was 2.58 m/s. When a 45.07-g iron bead fell freely from 0.55 m, the PE was 243.17 mJ and the velocity at impact was 3.28 m/s. The floor surface of the house model was divided into two types: a bare floor (BF), with a thickness of 2.92 mm, and a moss-graffiti floor (MF), with a thickness of 12.92 mm. That is, it was divided into a BHBF model without moss on six sides, a BHMF model with moss graffiti on the floor, a MHMF model with moss graffiti on six sides, and a MHBF model with moss graffiti (except for the floor). In experimental Case 1, as shown in Figure 4d, the MF model attenuated the noise by up to 34 dB in the high-frequency range of 2 to 4 kHz. As shown in Figure 4e, the MH model effectively attenuated the noise from low frequencies of approximately 200 to 1000 Hz to the high-frequency range of 2000 to 5000 Hz. This represents an attenuation of approximately 21 dB at 1250 Hz and 27 dB at 4000 Hz. In the case of the MHMF model, the noise-attenuation ability was greater in the high-frequency region, (Figure 4f). As shown in Figure 4g,h, using the heavy iron bead and a higher drop position to generate the sound, the MF model in the high-frequency region and the MH model in the low-frequency region achieved superior noise-damping performances. However, when a heavier iron bead fell from a greater height, there was a difference in the intensity of the noise level due to the increase in random collisions in the internal structure of the house model and the torsion of the path.

## 4. Discussion

The nature-inspired design of highly stacked moss-based structures provides a promising approach to the fabrication of sustainable materials that can replace artificial porous materials. This is the first demonstration of a moss-based noise- and shock-damping structure utilizing eco-friendly natural materials and its application as moss graffiti to a three-dimensional-house model. Moss dough ground to a millimetric scale can be an environmentally friendly sound-absorbing structural material that can be applied in any shape through a simple and effective manufacturing method. As a high-strength and sustainable structural material, it can be rapidly mass-produced, making it a strong competitor for artificial porous structures, such as polyurethane. In addition, by subjecting a three-dimensionally printed house model with a graffitied-moss-based composite to a sound-absorption test, we demonstrated that the moss-based material can be applied to external decorations, such as building walls, suggesting avenues for further empirical research into methods for effectively reducing noise pollution. We anticipate that moss-based porous structures with excellent mechanical and sound-absorption properties will soon play an important role in sustainable design, replacing environmentally destructive artificial porous foam. Therefore, it is possible to pursue a sustainable future by installing it inside and outside the building based on the improvement in the sound-absorption performance and compressive strength of moss-based sound-absorbing materials.

## Data Availability

Not applicable.
